# Diseases of the Spinal Cord

**Published:** 1886-07

**Authors:** 


**Diseases of the Spinal Cord.** By BYRON BRAMWELL, M. D., F. R. C. P., Edinburg, Lecturer on the Principles and Practice of Medicine, and on Medical Diagnosis in the Extra Academical School of Medicine, Edinburgh, etc. Second edition. New York: Wm. Wood & Co. 1886.

This book is one of the volumes contained in Wood's Library for 1886. That it will add greatly to the value of the series, no one will doubt who sees the book. The first edition of the work was well received by the profession, and it has been translated into the German, French and Russian languages, a fact which of itself attests the worth of the book. The chapter on the Anatomy and Physiology of the Spinal Segment is most excellent. Those on Pathology, classification, etc., are complete. The plates are good. All in all, this is one of the most complete books on the subject. Of itself, it is well worth one-fourth the price of the whole series of twelve.

